# Prevalence of malnutrition and the appropriateness of nutritional support among patients with hematologic malignancies: a GLIM-based study

**DOI:** 10.3389/fnut.2026.1939731

**Published:** 2026-09-22

**Authors:** Weiying Zhong, Xiamin Zhu, Fang Tang, Lan Xu

**Affiliations:** 1 Jiangsu Key Laboratory of Hematologic Diseases, Department of Hematology, The First Affiliated Hospital of Soochow University, Jiangsu Institute of Hematology, National Clinical Research Center for Hematologic Diseases, Suzhou, China; 2 Department of Nursing, The First Affiliated Hospital of Soochow University, Suzhou, China

**Keywords:** GLIM criteria, hematologic malignancies, malnutrition, nutritional risk, nutritional therapy

## Abstract

**Background:**

Malnutrition is common among patients with hematologic malignancies, but there are few clinical studies on disease-specific nutritional characteristics and the appropriateness of nutritional support.

**Methods:**

This cross-sectional study included 633 hospitalized patients with hematologic malignancies. Nutritional risk was assessed using the NRS-2002, malnutrition was diagnosed according to GLIM criteria, and the appropriateness of nutritional support was evaluated.

**Results:**

54.0% of patients were at nutritional risk, and 39.0% were diagnosed with malnutrition. Significant heterogeneity was observed across different disease subtypes, with multiple myeloma having the highest prevalence of nutritional risk (66.3%) and leukemia having the highest prevalence of malnutrition (44.9%). Female gender, diabetes, hypertension, elevated C-reactive protein, infection, and low serum albumin levels were independent risk factors associated with malnutrition. Only 15.6% of patients received nutritional support; fewer than one-third of malnourished patients received nutritional intervention, while 7.0% of well-nourished patients received nutritional support without clear indications.

**Conclusion:**

There is significant heterogeneity in the nutritional status of hospitalized patients with hematologic malignancies, and a marked mismatch exists between nutritional needs and nutritional support. Disease-specific nutritional management based on the GLIM assessment may improve the appropriateness of nutritional support and optimize clinical outcomes.

## 1 Introduction

Malignant hematologic tumors are a heterogeneous group of malignant diseases originating in the hematopoietic and lymphatic systems (1), with acute leukemia, lymphoma, and multiple myeloma being the most common (2). In recent years, with the continuous advancement of treatment modalities such as chemotherapy, targeted therapy, immunotherapy, and hematopoietic stem cell transplantation, patient survival outcomes have improved significantly (3–7). However, numerous challenges remain in the field of supportive care. Due to the combined effects of multiple factors—including disease-related metabolic abnormalities, systemic inflammatory responses, treatment-related toxicity, and inadequate dietary intake—patients with hematologic malignancies are highly susceptible to impaired nutritional status (8). Malnutrition, as an important yet often overlooked complication, can have multiple negative impacts on patient treatment, physical function, consumption of medical resources, and prognosis (9, 10).

Previous studies have shown (11) that the prevalence of malnutrition among patients with hematologic malignancies ranges from 15.0 to 64.7%, with significant variations depending on disease subtype and stage of treatment. Malnutrition not only increases the risk of infection, prolongs hospital stays, and raises healthcare costs, but also reduces patients’ tolerance to anticancer therapy, increasing the risk of treatment interruptions and dose adjustments, which ultimately affects remission rates and survival outcomes. Furthermore, malnutrition leads to decreased physical activity capacity, reduced quality of life, and increased psychological burden, thereby further exacerbating the overall disease burden.

Given the range of adverse effects caused by malnutrition, nutritional intervention offers clear benefits. A prospective controlled study conducted by Bargetzi et al. (12) demonstrated that individualized nutritional support can reduce mortality in cancer patients and effectively improve physical function and quality of life. Thus, malnutrition is one of the key modifiable factors affecting treatment outcomes and long-term prognosis in patients with hematologic malignancies. Early screening for nutritional risk and the implementation of individualized nutritional interventions have become integral components of comprehensive, lifelong management of hematologic malignancies.

Early identification of malnutrition is a crucial prerequisite for implementing targeted nutritional interventions. Currently, there are significant differences in the nutritional assessment tools and diagnostic criteria used in clinical practice, and there is marked heterogeneity in the criteria for determining malnutrition across different studies. This not only results in substantial variations in reported malnutrition prevalence rates among studies but may also lead to inadequate identification of populations at high risk for malnutrition (13–15). To achieve global standardization of malnutrition diagnostic criteria, the Global Leadership Initiative on Malnutrition (GLIM) published a consensus on malnutrition diagnosis in 2019 (16). The GLIM criteria employ a two-step diagnostic process: first, a nutritional risk screening is conducted, followed by a diagnosis based on a combination of phenotypic indicators (unintentional weight loss, low body mass index, and reduced muscle mass) and etiological indicators (reduced intake or absorption, inflammation, or disease burden) (16). In recent years, multiple studies have confirmed that the GLIM criteria can effectively identify malnutrition in cancer patients, and that a diagnosis of malnutrition is closely associated with adverse clinical outcomes such as infection, readmission, treatment toxicity, and mortality (17–19). As a result, the GLIM criteria have gradually become a widely recognized international tool for diagnosing malnutrition.

Nutritional support is an integral part of comprehensive cancer treatment. Relevant guidelines both domestically and internationally emphasize that for patients at nutritional risk or who are already malnourished, individualized nutritional interventions should be initiated as early as possible (20, 21). The selection of an appropriate nutritional support strategy, including oral nutritional supplementation (ONS), enteral nutrition (EN), and parenteral nutrition (PN), should primarily consider gastrointestinal function, while also considering patients’ ability to meet nutritional requirements through oral intake, swallowing function, aspiration risk, disease characteristics, anticipated duration of nutritional support, and overall clinical goals. In clinical practice, a combination of different nutritional modalities may be required when a single approach is insufficient to achieve nutritional targets, such as supplementing oral or EN with PN. Appropriate and standardized nutritional support helps maintain the body’s nutritional status, improve tolerance to anticancer therapy, reduce complications, and improve patients’ long-term prognosis. However, in the real world, a disparity between the current nutritional status and actual needs of hospitalized cancer patients remains widespread. On the one hand, many patients at nutritional risk or with malnutrition fail to receive timely nutritional support; on the other hand, some patients with good nutritional status receive nutritional therapy without clear indications. The coexistence of inadequate and excessive nutritional support not only affects the efficacy of nutritional therapy but may also increase the consumption of medical resources and the risk of related complications. Therefore, while assessing the prevalence of malnutrition, further evaluating the appropriateness of nutritional support is of great significance for improving standardized nutritional management and enhancing the quality of nutritional care for cancer patients.

At present, research on the nutritional status of patients with hematologic malignancies has largely focused on individual disease subtypes or specific stages of treatment, while studies that comprehensively examine the prevalence of malnutrition among hospitalized patients based on the GLIM criteria and simultaneously evaluate the appropriateness of nutritional support remain scarce. Although the GLIM criteria have been applied to diagnose malnutrition in hospitalized patients with hematologic malignancies (22), and nutritional support practices have been evaluated in general hospitalized populations (23), few studies have integrated these two aspects by examining the relationship between GLIM-defined malnutrition status and the appropriateness of nutritional interventions in patients with hematologic malignancies (24). Consequently, evidence regarding the concordance between nutritional status and nutritional management in this population remains limited.

Therefore, this study aims to use the GLIM criteria to investigate the prevalence of malnutrition among hospitalized patients with hematologic malignancies, analyze factors associated with malnutrition, and further assess the status of clinical nutritional support and evaluate the appropriateness of nutritional intervention protocols. The study aims to provide evidence-based support and clinical practice guidelines for optimizing comprehensive nutritional management plans for patients with hematologic malignancies, advancing precision nutritional support, and improving patient clinical outcomes.

## 2 Materials and methods

### 2.1 Participants

#### 2.1.1 Sample

This was a single-center cross-sectional observational study conducted in the Department of Hematology at the First Affiliated Hospital of Soochow University.

Patients with hematologic malignancies who were hospitalized between January 2025 and December 2025 were consecutively recruited. The inclusion criteria were as follows: participants were adults diagnosed with hematologic malignancies, including acute leukemia, chronic leukemia, lymphoma, multiple myeloma, and myelodysplastic syndromes, based on bone marrow cytology, histopathology, immunophenotyping, cytogenetic analysis, and/or molecular biological testing. Complete clinical and laboratory data were available. Participants were fully conscious, had normal cognitive function, exhibited no significant communication barriers, and were able to complete nutritional screening and GLIM-based malnutrition assessment. Written informed consent was obtained from all participants. The exclusion criteria were as follows: participants were excluded if they lacked a confirmed diagnosis of hematologic malignancy, received end-of-life palliative care with an expected survival time of less than 1 month, had severe comorbid conditions that could substantially affect nutritional status, had a history of psychiatric disorders, or were unable to complete the survey because of visual, hearing, cognitive, speech, or communication impairments.

#### 2.1.2 Sampling method

Eligible patients were recruited consecutively from the hematology department of the First Affiliated Hospital of Soochow University during the study period. Recruitment was based on hospital admission records and clinical diagnosis confirmation, ensuring that all participants met the inclusion criteria. Informed consent was obtained from each participant prior to enrollment.

### 2.2 Measures

#### 2.2.1 General information questionnaire for patients with hematologic malignancies

Based on an extensive literature review and in line with the study objectives, a general information questionnaire was independently designed. The questionnaire primarily covered sociodemographic and disease-related information, including age, sex, body mass index (BMI, kg/m^2^), educational level, place of residence, health insurance status, subtype of hematologic malignancy, diagnosed chronic diseases, laboratory test results, number of episodes of fever, duration of fever, occurrence of infection, type of nutritional support, length of hospital stay, and total hospitalization cost.

Patients were divided into two age groups: under 60 years and 60 years or older. BMI was categorized as underweight (BMI < 18.5), normal weight (18.5 ≤ BMI < 23.9), or overweight/obese (BMI ≥ 23.9), based on the WHO Expert Consultation recommendations for Asian populations (25). Educational attainment was classified as elementary school or below, high school/secondary education, or college and above. Place of residence was categorized as rural/town or county/city. Health insurance was categorized as basic medical insurance for urban employees, basic medical insurance for urban and rural residents, or self-funded medical insurance. Patients were classified by type of hematologic malignancy into the leukemia group, the lymphoma group, and the multiple myeloma group. Types of nutritional support included ONS, EN, and PN. In this study, ONS consisted of oral nutritional supplements prepared by the hospital nutrition department and commercially available oral enteral nutrition formulas. The latter involved intravenous administration of amino acids, fat emulsions, glucose, or total nutritional analogs (TNA).

Data on the type of hematologic malignancy, the number of diagnosed chronic conditions, laboratory test results, the number of episodes of fever, the duration of fever, the occurrence of infections, the type of nutritional support, the length of hospital stay, and total hospitalization costs were all obtained from medical records.

#### 2.2.2 Nutritional risk screening 2002

Nutritional risk was assessed using the Nutritional Risk Screening 2002 (NRS-2002). All patients were screened within 24 h after admission by trained nurses. The NRS-2002 consists of three components: nutritional status impairment (including BMI, recent weight loss, and decreased food intake), disease severity, and age adjustment. Each component was scored from 0 to 3 according to the standardized criteria, and an additional 1 point was added for patients aged ≥70 years. The total score ranges from 0 to 7, and patients with an NRS-2002 score ≥3 were considered at nutritional risk (26).

#### 2.2.3 GLIM criteria

In this study, patients were classified into three groups according to the GLIM criteria: no malnutrition, moderate malnutrition and severe malnutrition (16). The GLIM framework assesses the risk of malnutrition using phenotypic and alethiological criteria. Phenotypic criteria include unintentional weight loss, a low BMI and low muscle mass. Phenotypic criteria include unintentional weight loss, a low BMI and low muscle mass. Low BMI is defined using age-specific cut-off values for Asian populations: <20.0 kg/m^2^ for those aged ≥70 years, and <18.5 kg/m^2^ for those under 70 years. Severe low BMI is defined as: <17.8 kg/m^2^ for those aged ≥70 years, and <17.0 kg/m^2^ for those under 70 years (27). Low muscle mass is defined in accordance with the GLIM guidelines (28), using the recommended cut-off values for BIA measurements: ASMI < 7.0 kg/m^2^ for men and < 5.7 kg/m^2^ for women. Severe low muscle mass is further defined as: ASMI < 5.2 kg/m^2^ for men and < 4.8 kg/m^2^ for women (29). The definition of low muscle mass is based on the GLIM guidelines (28), using the recommended cut-off values for BIA measurements: ASMI < 7.0 kg/m^2^ for men and < 5.7 kg/m^2^ for women. Severe low muscle mass is further defined as: ASMI < 5.2 kg/m^2^ for men and < 4.8 kg/m^2^ for women (29). Weight loss is defined according to GLIM criteria: a weight loss of more than 5% over the past 6 months, or a weight loss of more than 10% regardless of the time frame. Weight loss is defined according to GLIM criteria: a weight loss of more than 5% over the past 6 months, or a weight loss of more than 10% regardless of the time frame. Severe weight loss is defined as a weight loss of more than 10% over 6 months, or a weight loss of more than 20% regardless of the time frame. Severe weight loss is defined as a weight loss of more than 10% over 6 months, or a weight loss of more than 20% regardless of the time frame.

#### 2.2.4 Appendicular skeletal muscle mass index

Appendicular skeletal muscle mass (ASM) index (ASMI) was estimated using the intermediate prediction equation proposed by Hsu et al. (30), which was selected because it is based on routinely available variables and showed the highest correlation with measured muscle mass among feasible equations in the original study. ASMI was calculated by dividing estimated ASM by height squared. In this study, ASM was not directly measured by bioelectrical impedance analysis (BIA) but was instead estimated using a previously validated predictive equation, with BIA as the reference method, based on variables routinely available in administrative and clinical datasets, as follows: ASM = −6.747 - 0.054 × age (years) – 2.234 × sex (male = 0, female = 1) + 0.093 × height (cm) + 0.210 × weight (kg), by height squared (30).

### 2.3 Statistical analysis

All statistical analyses were performed using SPSS 27.0 software. Descriptive statistics were calculated for all variables. Categorical variables were expressed as frequencies/percentages and compared using two-sided chi-square tests or Fisher’s exact tests. Continuous data were reported as mean ± standard deviation (SD) or median [interquartile range (IQR)] and analyzed using independent t-tests or the Mann–Whitney U test. Where necessary, continuous variables were dichotomized or grouped. Univariate analyses were conducted first to explore the impact of associated factors on malnutrition; finally, multivariate logistic regression models were established. A two-tailed *p*-value < 0.05 was considered statistically significant.

### 2.4 Ethical considerations

The Ethics Committee of the First Affiliated Hospital of Soochow University approved this study (No. 2026144). Informed consent was obtained from all participants.

## 3 Results

### 3.1 General characteristics

This study included a total of 633 hospitalized patients with hematologic malignancies. Among them, 342 patients (54.0%) were identified as being at nutritional risk, defined as an NRS-2002 score of ≥3. According to the GLIM criteria, which require both nutritional risk (NRS-2002 score ≥ 3) and the fulfillment of at least one phenotypic and one etiological criterion, a total of 247 patients (39.0%) were diagnosed with malnutrition. Among them, 507 (80.1%) were under 60 years of age; 317 (50.1%) were male, 248 (39.2%) resided in urban areas, and 191 (30.2%) did not have a fever during hospitalization in Tables 1–4.

**Table 1:** Nutritional risk identified by NRS-2002 among patients with different hematological malignancies.

Hematological malignancy type	Nutritional risk (*N* = 342)	No nutritional risk (*N* = 291)	Total (*N* = 633)	Proportion of nutritional risk (%)	χ^2^	*p*
Leukemia	157(58.8)	110(41.2)	267	58.8	14.277	0.001
Multiple myeloma	55(66.3)	28(33.7)	83	66.3		
Lymphoma	131(46.3)	152(53.7)	283	46.3		
Overall	342	291	633	54.0		

**Table 2:** Malnutrition assessed by GLIM among patients with different hematological malignancies.

Hematological malignancy type	Malnutrition (*N* = 247)	Non-malnutrition (*N* = 386)	Total (*N* = 633)	Proportion of malnutrition (%)	χ^2^	*p*
Leukemia	120(44.9)	147(55.1)	267	44.9	7.021	0.030
Multiple myeloma	27(32.5)	56(67.5)	83	32.5		
Lymphoma	100(35.3)	183(64.7)	283	35.3		
Overall	247	386	633	39.0		

**Table 3:** Malnutrition assessed by GLIM among patients with different hematological malignancies.

Hematological malignancy type	Moderate malnutrition (*N* = 158)	Severe malnutrition (*N* = 89)	Non-malnutrition (*N* = 386)	χ^2^	*p*
Leukemia	77(48.7)	43(48.3)	147(38.1)	7.132	0.129
Multiple myeloma	18(11.4)	9(10.1)	56(14.5)		
Lymphoma	63(39.9)	37(41.6)	183(47.4)		

**Table 4:** The univariate model of malnutrition in patients with hematologic malignancies.

Characteristic	Total (*N* = 633)	Moderate malnutrition (*N* = 158)	Severe malnutrition (*N* = 89)	Non-malnutrition (*N* = 386)	H/χ^2^	*p*
Gender					16.690	<0.001
Male	317(50.1)	69(43.7)	31(34.8)	217(56.2)		
Female	316(49.9)	89(56.3)	58(65.2)	169(43.8)		
Age (years)					6.359	0.042
<60	507(80.1)	133(84.2)	77(86.5)	297(76.9)		
≥60	126(19.9)	25(15.8)	12(13.5)	89(23.1)		
Education					10.190	0.117
Elementary school and below	168(26.5)	36(22.8)	18(20.2)	114(29.5)		
High school/secondary school	250(39.5)	63(39.9)	43(48.3)	144(37.3)		
Post-secondary	143(22.6)	41(25.9)	14(15.7)	88(22.8)		
College and above	65(10.3)	18(11.4)	14(15.7)	40(10.4)		
Place of residence					6.589	0.037
Urban/County	248(39.2)	59(44.2)	25(39.5)	164(49.7)		
Town/Rural	385(60.8)	99(55.8)	64(60.5)	222(50.3)		
Medical insurance					4.331	0.363
UEMBI	376(59.4)	99(62.7)	57(64.0)	220(57.0)		
URRBMI	230(36.3)	52(32.9)	31(34.8)	147(38.1)		
Self-financed	27(4.3)	7(4.4)	1(1.1)	19(4.9)		
Fever					13.769	0.008
0	191(30.2)	47(29.7)	29(32.6)	115(29.8)		
1–3	99(15.6)	35(22.2)	19(21.3)	45(11.7)		
>3	343(54.2)	76(48.1)	41(46.1)	226(58.5)		
Duration of fever (days)	0.0(0.0,2.0)	0.0(0.0,3.0)	0.0(0.0,3.0)	0.0(0.0,2.0)	6.321	0.042
Infection	166(26.2)	45(28.5)	36(40.4)	85(22.0)	13.250	0.001
Hypertension	275(43.4)	73(46.2)	53(59.6)	149(38.6)	13.571	0.001
Diabetes	131(20.7)	34(21.5)	37(41.6)	60(15.5)	29.943	<0.001
Other complications	145(22.9)	43(27.2)	28(31.5)	74(19.2)	8.399	0.015
Chronic disease					3.760	0.153
0–2	394(62.2)	104(65.8)	61(68.5)	229(59.3)		
>2	239(37.8)	54(34.2)	28(31.5)	157(40.7)		
Length of hospital stay (days)	13.0(6.0,24.0)	19.0(9.0,31.0)	20.0(9.0,33.0)	11.0(6.0,19.0)	48.179	<0.001
Total Hospitalization Costs(ten thousand yuan)	5.6(3.1,8.7)	8.8(3.5,11.0)	8.2(4.2,19.1)	5.0(2.8,7.0)	73.479	<0.001
Hemoglobin	72.0(63.5,86.0)	72.5(63.0,86.5)	70.0(57.0,86.0)	73.0(65.0,88.0)	6.808	0.033
Platelet count	113.0(52.0,220.0)	115.5(59.5,229.3)	173.0(73.5,261.0)	96.0(46.8,210.0)	13.862	0.001
C-reactive protein	7.3(2.1,34.5)	6.3(2.9,32.4)	19.1(4.6,50.1)	6.5(1.8,31.3)	12.827	0.002
Albumin	37.4(33.7,40.4)	37.8(34.0,40.5)	35.0(31.2,39.2)	37.6(34.3,40.5)	18.135	<0.001
Prealbumin	231.1(182.3,278.0)	241.8(188.6,292.1)	214.3(183.4,276.0)	223.4(179.7,275.1)	7.029	0.030
BMI (kg/m^2^)					2.863	0.581
<18.5	196(31.0)	47(29.7)	24(27.0)	125(32.4)		
18.5–23.9	266(42.0)	68(43.0)	44(49.4)	154(39.9)		
≥23.9	171(27.0)	43(27.2)	21(23.6)	107(27.7)		
Hematological malignancy type					7.132	0.129
Leukemia	267(42.2)	77(48.7)	43(48.3)	147(38.1)		
Multiple myeloma	83(13.1)	18(11.4)	9(10.1)	56(14.5)		
Lymphoma	283(44.7)	63(39.9)	37(41.6)	183(47.4)		
Nutritional intervention	99(15.6)	35(22.2)	37(41.6)	27(7.0)	65.345	<0.001

### 3.2 Nutritional risk screening results

A total of 633 patients with hematologic malignancies were included in the study, of whom 342 were at nutritional risk (NRS-2002 score ≥ 3), representing a nutritional risk prevalence of 54.0%. A statistically significant difference was found in the prevalence of nutritional risk among patients with different disease types (χ^2^ = 14.277, *p* = 0.001). Patients with multiple myeloma had the highest prevalence of nutritional risk (66.3%), followed by those with leukemia (58.8%), while patients with lymphoma had the lowest prevalence (46.3%) in Tables 1–3.

### 3.3 Malnutrition outcomes

According to the GLIM malnutrition diagnostic criteria, there must be a nutritional risk (NRS-2002 score ≥ 3), and at least one phenotypic indicator and one etiological indicator. A total of 247 patients were diagnosed with malnutrition, and the overall prevalence of malnutrition was 39.0%. There were significant differences in the prevalence of malnutrition among patients with different disease types (χ^2^ = 7.021, *p* = 0.030). The prevalence of malnutrition was the highest in leukemia patients (44.9%), followed by lymphoma patients (35.3%) and myeloma patients (32.5%). Further analysis revealed that 158 patients were diagnosed with moderate malnutrition (64.0%), and 89 patients were diagnosed with severe malnutrition (36.0%). There was no statistically significant difference in the composition ratios of moderate and severe malnutrition among patients with different disease types (χ^2^ = 7.132, *p* = 0.129) in Tables 1–3.

### 3.4 Factors associated with malnutrition

The results of the univariate analysis showed that gender, age, place of residence, number of fever episodes, duration of fever, infection, hypertension, diabetes, other comorbidities, length of hospital stay, total hospitalization costs, hemoglobin, platelet count, C-reactive protein, albumin, prealbumin were significantly associated with the occurrence of malnutrition (all *p* < 0.05) in Tables 1–3.

Multinomial logistic regression models were constructed using malnutrition categories as the dependent variable and variables that were statistically significant in the univariate analysis as independent variables. A multiclass Logistic regression analysis was conducted with patients without malnutrition as the reference group. The results showed that the length of hospital stay (OR = 1.038, 95% CI: 1.012–1.063, *p* = 0.003), total hospitalization cost (OR = 1.198, 95% CI: 1.112–1.290, *p* < 0.001), age < 60 years (OR = 2.301, 95% CI: 1.303–4.062, *p* = 0.004), hypertension (OR = 1.976, 95% CI: 1.272–3.071, *p* = 0.002), and diabete count (OR = 2.055, 95% CI: 1.212–3.485, *p* = 0.008) were independent risk factors for moderate malnutrition, and the risk of moderate malnutrition in men was lower than that in women (OR = 0.647, 95% CI: 0.429–0.975, *p* = 0.037). Further analysis of the influencing factors of severe malnutrition revealed that hospitalization costs (OR = 1.543, 95% CI: 1.392–1.711, *p* < 0.001), platelet count (OR = 1.003, 95% CI: 1.001–1.005, *p* = 0.014), C-reactive protein level (OR = 1.013, 95% CI: 1.000–1.026, *p* = 0.044), age < 60 years (OR = 4.998, 95% CI: 1.859–13.438, *p* = 0.001), infection (OR = 2.698, 95% CI: 1.323–5.501, *p* = 0.006), coexisting hypertension (OR = 3.913, 95% CI: 2.004–7.640, *p* < 0.001), and coexisting diabetes (OR = 5.618, 95% CI: 2.827–11.163, *p* < 0.001) were all independent risk factors for severe malnutrition; while higher albumin levels (OR = 0.858, 95% CI: 0.796–0.924, *p* < 0.001), being male (OR = 0.506, 95% CI: 0.270–0.947, *p* = 0.033), and living in urban areas (OR = 0.367, 95% CI: 0.179–0.751, *p* = 0.006) were protective factors in Supplementary Table 1.

### 3.5 Status of nutritional therapy

Among the 633 patients, only 99 received nutritional intervention, with a total intervention rate of 15.6%. There were significant differences in the nutritional intervention rates among patients with different disease types (χ^2^ = 24.051, *p* < 0.001). Among them, the nutritional intervention rate was the highest in leukemia patients (22.8%), followed by myeloma patients (18.1%), and the lowest in lymphoma patients (8.1%) in Supplementary Table 2. Among the 247 malnourished patients, only 72 received nutritional support, with a support rate of 29.1%. There were significant differences in the proportion of patients receiving nutritional support based on their nutritional status (χ^2^ = 10.175, *p* = 0.001). Among patients with moderate malnutrition, 35 cases (22.2%) received nutritional support, and among those with severe malnutrition, 37 cases (41.6%) received nutritional support, while 27 cases (7.0%) of patients without malnutrition also received nutritional support in Supplementary Table 3. The results showed that most malnourished patients did not receive nutritional support, while some patients without malnutrition received nutritional intervention. In terms of specific nutritional support methods, ONS were the most used nutritional intervention measure. There were significant differences in the proportions of patients receiving ONS, enteral nutrition, and parenteral nutrition between patients with and without malnutrition (all *p* < 0.05), with higher proportions observed among malnourished patients in Supplementary Table 4.

## 4 Discussion

### 4.1 Heterogeneity of nutritional risk and malnutrition among hematologic malignancies

This study found that among hospitalized patients with hematologic malignancies, the prevalence of nutritional risk and malnutrition was high, respectively. There was significant heterogeneity in the prevalence of nutritional risk and malnutrition across different disease subtypes: patients with multiple myeloma had the highest prevalence of nutritional risk, but their prevalence of malnutrition was lower than that of patients with leukemia. Although the prevalence of nutritional risk was not the highest among leukemia patients, they exhibited the highest prevalence of malnutrition. These results suggest that nutritional risk and malnutrition are not entirely synonymous concepts but rather reflect different stages of impaired nutritional status, and that patterns of nutritional impairment may vary across different types of hematologic malignancies. This discrepancy may be related to the differences in nutritional status reflected by the NRS-2002 and GLIM. The NRS-2002 is primarily used to identify the risk of future malnutrition (26), whereas the GLIM emphasizes existing nutritional deficiencies and tissue wasting (16).

The pattern of nutritional impairment in patients with leukemia is characterized by rapid metabolic deterioration; while their nutritional risk is not the highest, they have the highest prevalence of malnutrition. This may be due to the aggressive nature of leukemia and the intensive treatment required during hospitalization. The rapid proliferation of leukemia cells leads to protein breakdown and gluconeogenesis, increasing the body’s resting energy expenditure (31); at the same time, induction chemotherapy often causes side effects such as nausea, vomiting, mucositis, and diarrhea, which in turn lead to loss of appetite and weight loss (32). Together, these factors significantly reduce patients’ dietary intake while increasing metabolic demands, accelerating the progression from nutritional risk to overt malnutrition. Rapid disease progression and ongoing intensive treatment deprive patients of an adequate window for nutritional recovery (33), making them particularly vulnerable to rapid deterioration of their nutritional status. The pattern of nutritional impairment in lymphoma is driven by disease heterogeneity, with relatively low levels of nutritional risk and malnutrition. Among hematologic malignancies, lymphoma has a relatively slow disease progression; unless there is gastrointestinal involvement or advanced disease, oral intake and gastrointestinal function are typically well preserved (34). Some patients undergo cyclic chemotherapy, during which their appetite recovers between treatment cycles, and their dietary intake and nutritional status remain good. These factors partly explain the relatively low prevalence of nutritional impairment observed in this study; however, in real-world clinical settings, clinicians must remain vigilant regarding the higher nutritional risk faced by patients with aggressive or advanced-stage lymphoma. The nutritional impairment pattern in multiple myeloma is characterized by chronic inflammation vulnerability, which has the highest prevalence of nutritional risk but not the highest prevalence of malnutrition. This pattern reflects the chronic nature of nutritional impairment in multiple myeloma. Persistent systemic inflammation, driven by cytokines such as interleukin-6, promotes protein catabolism, skeletal muscle loss, and cancer-related fatigue (35); meanwhile, bone-destructive disease, chronic pain, anemia, and renal impairment further reduce physical activity levels and functional capacity (36). These factors collectively lead to persistent impairment of nutritional status and increase patients’ likelihood of being at nutritional risk, resulting in a high prevalence of nutritional risk. However, because the deterioration of nutritional status typically progresses slowly, many patients may still be in the nutritional risk stage and have not yet reached the stage of malnutrition. This finding further indicates that nutritional risk and malnutrition represent different stages of impaired nutritional status, rather than mutually exclusive clinical conditions.

The above findings are significant for clinical nutrition management. A single nutrition management strategy is unlikely to fully meet the diverse nutritional needs of patients with various hematologic malignancies. Personalized nutrition care plans should be developed based on disease-specific patterns of nutritional impairment. Incorporating nutritional assessment into routine care evaluations in the future will help identify patients at high risk of malnutrition earlier and facilitate timely, personalized nutritional interventions.

### 4.2 Factors associated with malnutrition

Results of the multivariate analysis showed that moderate malnutrition was associated with gender, prolonged hospital stays, increased total hospitalization costs, and comorbidities; severe malnutrition was further closely associated with infection, elevated C-reactive protein levels, and decreased albumin levels. These findings suggest that malnutrition in patients with hematologic malignancies is not solely caused by inadequate food intake, but rather results from the combined effects of disease severity, inflammatory burden, and comorbidities.

The GLIM Consensus identifies inflammation as a key etiological factor in malnutrition (37). In this study, elevated C-reactive protein was independently associated with severe malnutrition, whereas higher serum albumin levels were independently protective, further supporting the role of inflammation in malnutrition. Inflammatory responses promote skeletal muscle protein breakdown, increase resting energy expenditure, and suppress appetite through cytokine release, ultimately leading to weight loss and muscle loss (38, 39). Notably, these metabolic alterations may precede changes in body weight, indicating that malnutrition can already be present despite a normal BMI. Infection was another independent risk factor for severe malnutrition. Patients with hematologic malignancies are particularly susceptible to infection because of immune dysfunction and chemotherapy-induced bone marrow suppression. Infection further increases energy expenditure, reduces appetite, and aggravates gastrointestinal symptoms, thereby accelerating nutritional deterioration (22). Moreover, malnutrition and infection may reinforce each other, forming a vicious cycle that further compromises nutritional status. Both diabetes and hypertension were identified as independent risk factors for malnutrition, with stronger associations observed in patients with severe malnutrition. Although chronic metabolic diseases have traditionally been linked to overweight and obesity, emerging evidence suggests that patients with diabetes may simultaneously experience muscle loss and inadequate nutrient intake, a condition referred to as metabolic sarcopenia (40). These findings suggest that relying solely on body weight or BMI may underestimate nutritional impairment in patients with chronic metabolic diseases.

Female sex was also independently associated with malnutrition, consistent with previous studies. Women generally have lower skeletal muscle mass than men, and hormonal differences may reduce their capacity to withstand chronic inflammation and cancer-related hypermetabolism, increasing susceptibility to muscle loss (41). In addition, greater sensitivity to gastrointestinal symptoms may contribute to reduced food intake and accelerate nutritional deterioration. Living in rural areas was identified as an independent risk factor for malnutrition, suggesting that socioeconomic and healthcare disparities may influence nutritional outcomes (42). Compared with urban patients, rural patients often have lower health literacy, limited access to nutritional services, and delayed nutritional assessment, increasing the likelihood of malnutrition at hospital admission. These findings highlight the need for healthcare professionals to strengthen nutritional screening, health education, and continuity of nutritional care for rural patients.

The findings of this study suggest that a comprehensive nutritional assessment system incorporating disease heterogeneity and risk stratification should be established for patients with hematologic malignancies. In addition to routine nutritional screening, healthcare professionals should implement dynamic nutritional monitoring based on laboratory indicators, disease-related factors, and demographic characteristics. Individualized nutritional interventions should then be tailored to the nutritional impairment patterns of different hematologic malignancies to improve the early identification of malnutrition and optimize precision nutritional management.

### 4.3 Gap between nutritional needs and nutritional support

The most notable finding of this study is the significant disconnect between nutritional needs and nutritional support. Although 39.0% of patients met the GLIM criteria for malnutrition, only 15.6% of patients received nutritional intervention. Even among patients with moderate and severe malnutrition, the proportions receiving nutritional support were only 22.2 and 41.6%, respectively, suggesting that nutritional issues in patients with hematologic malignancies remain under-identified and under-treated. This phenomenon reflects a significant screening-intervention gap in the field of hematologic oncology. Nutritional management should follow a closed-loop process of screening—assessment—diagnosis—intervention—re-evaluation (43); however, in clinical practice, nutritional screening often stops at the risk identification stage, lacking follow-up referral and ongoing intervention mechanisms. Consequently, screening results are not effectively translated into clinical action.

Notably, 7.0% of patients without malnutrition also received nutritional support. This phenomenon suggests that decisions regarding nutritional support in clinical practice are not entirely based on objective nutritional assessment results but may be influenced by factors such as disease severity, treatment-related adverse reactions, risk of reduced food intake, and clinical judgment. For some patients about to undergo intensive chemotherapy, hematopoietic stem cell transplantation, or those with significant gastrointestinal symptoms, the clinical team may adopt a preventive nutritional support strategy, leading to premature nutritional intervention for some patients who have not yet met the diagnostic criteria for malnutrition (44). Furthermore, this study found that ONS were the primary form of nutritional support, while the use of EN and PN was relatively low (45). This suggests that nutritional support currently remains primarily supplementary, and that intensive nutritional therapy for patients with moderate-to-severe malnutrition has not yet been fully implemented.

This finding further underscores the importance of establishing a standardized nutrition management system. As the primary implementers of nutrition risk screening and ongoing monitoring, nursing staff should conduct continuous nutrition assessments using standardized screening tools and the GLIM assessment process, and promptly refer high-risk patients to the nutrition support team. This will facilitate the precise allocation of nutrition support resources to patients who truly need them and improve the timeliness and appropriateness of nutritional interventions.

### 4.4 Limitations and strengths

First, this was a single-center cross-sectional study conducted at a hematology center in China, which may limit the generalizability of the findings to other institutions and populations. Multicenter prospective studies across diverse clinical settings are needed to validate these findings. Second, the cross-sectional design precludes causal inference regarding the relationships among inflammatory status, clinical characteristics, and malnutrition. Longitudinal studies are warranted to clarify changes in nutritional status throughout treatment and evaluate the impact of early nutritional intervention on clinical outcomes. Third, skeletal muscle mass was estimated using a validated predictive equation rather than measured directly by bioelectrical impedance analysis or computed tomography. Although this approach has demonstrated good agreement with direct measurements when body composition assessment is not feasible, some degree of misclassification cannot be excluded.

Despite these limitations, this study has several notable strengths. To our knowledge, it is the first to apply the GLIM criteria to comprehensively evaluate hospitalized patients with different hematologic malignancies by examining the prevalence of malnutrition, associated risk factors, and the appropriateness of nutritional support. By covering the continuum from nutritional risk screening and malnutrition diagnosis to nutritional intervention, this study identified an important gap between nutritional needs and current clinical practice. These findings provide evidence to support the development of disease-specific nutritional management pathways and multidisciplinary nutritional care for patients with hematologic malignancies.

## 5 Conclusion

This study demonstrates marked heterogeneity in nutritional risk, GLIM-defined malnutrition, and nutritional support among hospitalized patients with hematologic malignancies, indicating that a uniform nutritional management strategy may not adequately meet the needs of different disease subtypes. Importantly, a substantial mismatch exists between nutritional needs and nutritional support: many malnourished patients do not receive nutritional intervention, whereas some well-nourished patients receive nutritional support without clear indications. These findings highlight a persistent gap between nutritional assessment and individualized intervention. Future clinical practice should incorporate GLIM-based malnutrition diagnosis into routine nutritional assessment and establish standardized nutritional care pathways integrating screening, diagnosis, intervention, and follow-up. Such approaches may improve the appropriateness of nutritional support and ultimately optimize clinical outcomes in patients with hematologic malignancies.

## Data Availability

The raw data supporting the conclusions of this article will be made available by the authors, without undue reservation.
