# Supplementary material for: Prevalence of malnutrition and the appropriateness of nutritional support among patients with hematologic malignancies: a GLIM-based study
Source: Front Nutr. 2026 Sep 22;13:1939731. doi: 10.3389/fnut.2026.1939731 (PMC13638393; doi:10.3389/fnut.2026.1939731)
Supplement: Supplementary file 1 [file Table_1.DOCX]

| **Supplementary Table 1** Multivariate logistic regression analysis of malnutrition in patients with hematologic malignancies | | | | | | | |
| --- | --- | --- | --- | --- | --- | --- | --- |
| Characteristic |  | β | SE | Wald χ2 | OR | 95%CI | *P* |
| ***Moderate malnutrition VS Non-malnutrition*** |  |  |  |  |  |  |  |
| ***Constant*** |  | -3.033 | 1.093 | 7.698 | - | - | 0.006 |
| ***Length of hospital stay*** |  | 0.037 | 0.013 | 8.645 | 1.038 | 1.012~1.063 | 0.003 |
| ***Total Hospitalization Costs*** |  | 0.181 | 0.038 | 22.681 | 1.198 | 1.112~1.290 | <0.001 |
| ***Gender*** | Male | -0.436 | 0.209 | 4.338 | 0.647 | 0.429~0.975 | 0.037 |
| ***Age*** | ＜60 | 0.833 | 0.290 | 8.255 | 2.301 | 1.303~4.062 | 0.004 |
| ***Complication*** | Hypertension | 0.681 | 0.225 | 9.188 | 1.976 | 1.272~3.071 | 0.002 |
|  | Diabetes | 0.720 | 0.269 | 7.143 | 2.055 | 1.212~3.485 | 0.008 |
| ***Severe malnutrition VS Non-malnutrition*** |  |  |  |  |  |  |  |
| ***Constant*** |  | -2.096 | 1.671 | 1.573 | - | - | 0.210 |
| ***Total Hospitalization Costs*** |  | 0.434 | 0.053 | 67.788 | 1.543 | 1.392~1.711 | <0.001 |
| ***Platelets*** |  | 0.003 | 0.001 | 6.033 | 1.003 | 1.001~1.005 | 0.014 |
| ***C-reactive protein*** |  | 0.013 | 0.006 | 4.072 | 1.013 | 1.000~1.026 | 0.044 |
| ***Albumin*** |  | -0.153 | 0.038 | 16.239 | 0.858 | 0.796~0.924 | <0.001 |
| ***Gender*** | Male | -0.682 | 0.320 | 4.539 | 0.506 | 0.270~0.947 | 0.033 |
| ***Age*** | ＜60 | 1.609 | 0.505 | 10.166 | 4.998 | 1.859~13.438 | 0.001 |
| ***Place of residence*** | Urban/County | -1.003 | 0.366 | 7.528 | 0.367 | 0.179~0.751 | 0.006 |
| ***Infection*** |  | 0.992 | 0.364 | 7.454 | 2.698 | 1.323~5.501 | 0.006 |
| ***Complication*** | Hypertension | 1.364 | 0.341 | 15.976 | 3.913 | 2.004~7.640 | <0.001 |
|  | Diabetes | 1.726 | 0.350 | 24.268 | 5.618 | 2.827-11.163 | <0.001 |
