# Supplementary material for: Prevalence of malnutrition and the appropriateness of nutritional support among patients with hematologic malignancies: a GLIM-based study
Source: Front Nutr. 2026 Sep 22;13:1939731. doi: 10.3389/fnut.2026.1939731 (PMC13638393; doi:10.3389/fnut.2026.1939731)
Supplement: Supplementary file 2 [file Table_2.DOCX]

| **Supplementary Table 2** Nutritional interventions for patients with different types of hematological malignancies | | | | | | |
| --- | --- | --- | --- | --- | --- | --- |
| Hematological malignancy type | Nutritional  interventions  (N = 99) | No nutritional  interventions  (N = 534) | Total  (N = 633) | Proportion of nutritional  interventions (%) | χ2 | *P* |
| Leukemia | 61(22.8) | 206(77.2) | 267 | 22.8 | 24.051 | <0.001 |
| Multiple myeloma | 15(18.1) | 68(81.9) | 83 | 18.1 |  |  |
| Lymphoma | 23(8.1) | 260(91.9) | 283 | 8.1 |  |  |
| Overall | 99 | 534 | 633 | 15.6 |  |  |
