# Supplementary material for: Prevalence of malnutrition and the appropriateness of nutritional support among patients with hematologic malignancies: a GLIM-based study
Source: Front Nutr. 2026 Sep 22;13:1939731. doi: 10.3389/fnut.2026.1939731 (PMC13638393; doi:10.3389/fnut.2026.1939731)
Supplement: Supplementary file 3 [file Table_3.DOCX]

| **Supplementary Table 3** Nutritional interventions by malnutrition severity | | | | | | |
| --- | --- | --- | --- | --- | --- | --- |
| Types of Malnutrition | Nutritional  interventions  (N = 72) | No nutritional  interventions  (N = 175) | Total  (N = 247) | Proportion of nutritional risk (%) | χ2 | *P* |
| Moderate malnutrition | 35(22.2) | 123(77.8) | 158 | 22.2 | 10.175 | 0.001 |
| Severe malnutrition | 37(41.6) | 52(58.4) | 89 | 41.6 |  |  |
| Overall | 72 | 175 | 247 | 29.1 |  |  |
