# Supplementary material for: Prevalence of malnutrition and the appropriateness of nutritional support among patients with hematologic malignancies: a GLIM-based study
Source: Front Nutr. 2026 Sep 22;13:1939731. doi: 10.3389/fnut.2026.1939731 (PMC13638393; doi:10.3389/fnut.2026.1939731)
Supplement: Supplementary file 4 [file Table_4.DOCX]

| **Supplementary Table 4** Distribution of Different Nutritional Support Modalities | | | | | |
| --- | --- | --- | --- | --- | --- |
| Types of Nutritional Support | Moderate malnutrition (N = 39) | Severe malnutrition  (N = 21) | Non-malnutrition  (N = 29) | χ2 | *P* |
| ONS | 27(17.1) | 30(33.7) | 22(5.7) | 49.473 | <0.001 |
| EN | 9(5.7) | 7(7.9) | 4(1.0) | 14.960 | 0.001 |
| PN | 3(1.9) | 9(10.1) | 3(0.8) | 18.765 | <0.001 |
